# Identification of excretory and secretory proteins from *Haemonchus contortus* inducing a Th9 immune response in goats

**DOI:** 10.1186/s13567-022-01055-8

**Published:** 2022-05-21

**Authors:** Meng Liang, Mingmin Lu, Muhammad Tahir Aleem, Yang Zhang, Mingyue Wang, Zhaohai Wen, Xiaokai Song, Lixin Xu, Xiangrui Li, Ruofeng Yan

**Affiliations:** MOE Joint International Research Laboratory of Animal Health and Food Safety, College of Veterinary Medicine, Nanjing Agricultural University, Nanjing, 210095 Jiangsu China

**Keywords:** *Haemonchus contortus*, Th9 immune response, proteomics, binding molecules, HcDR, HcGATA

## Abstract

Th9 cells have been shown to play crucial roles in anti-parasite immunity, pathogenic microbe infection, and allergy. Previous studies have demonstrated that *Haemonchus contortus* excretory and secretory proteins (HcESPs) induce the proliferation of Th9 cells and alter the transcriptional level of IL-9 as well as its related pathways in the Th9 immune response after infection. However, the exact molecule(s) in HcESPs inducing the Th9 immune response is not yet known. In this study, flow cytometry, co-immunoprecipitation (Co-IP) and shotgun liquid chromatography tandem-mass spectrometry (LC–MS/MS) were used, and a total of 218 proteins from HcESPs that might interact with goat Th9 cells were identified. By in vitro culture of Th9 cells with HcESPs, 40 binding proteins were identified. In vivo, 38, 47, 42 and 142 binding proteins were identified at 7, 15, 35 and 50 days post-infection (dpi), respectively. Furthermore, 2 of the 218 HcESPs, named DNA/RNA helicase domain containing protein (HcDR) and GATA transcription factor (HcGATA), were confirmed to induce the proliferation of Th9 cells and promote the expression of IL-9 when incubated with goat peripheral blood mononuclear cells (PBMCs). This study represents a proteomics-guided investigation of the interactions between Th9 cells and HcESPs. It provides a new way to explore immunostimulatory antigens among HcESPs and identifies candidates for immune-mediated prevention of *H. contortus* infection.

**Supplementary Information:**

The online version contains supplementary material available at 10.1186/s13567-022-01055-8.

## Introduction

*Haemonchus contortus* is one of the most important parasitic nematodes that mainly infect small ruminants (sheep and goat). Infection with *H. contortus* results in haemorrhagic gastritis with clinical symptoms, including anaemia, diarrhoea, weight loss, and even death in severe cases [1]. Haemonchosis is widely distributed around the world and causes enormous economic losses in cattle, sheep, and goat breeding industries. With increasing concerns about food safety and the relevance of anthelmintic resistance [2–4], it has been recognized that the development of effective and sustainable control strategies, including novel vaccines, is necessary to enhance the host anti-parasite immune response.

At present, parallel to that against other gastrointestinal (GI) nematodes, research on goat cellular immunity against *H. contortus* infection is mainly focused on the type II immune response represented by eosinophilia, mastocytosis and increased IgG1, IgG4, and IgE production; when Th1-type cells are persistent, they are associated with chronic health problems [5]. Th9 cells, which mainly produce the cytokine IL-9, have been implicated in allergic inflammation, autoimmune disease, antitumour immunity, and anti-parasite immunity [6–10]. In the early stage of parasitic infection, IL-9 is released from Th9 cells to initiate host immune responses [11]. At the same time, the numbers of tissue mast cells and basophils increase significantly, as do the levels of IgE and IgG antibodies, which stimulate and strengthen intestinal contractions, thereby expelling parasites [11]. Grencis et al. demonstrated that the helminth burden was proportional to the secretion of IL-9 by cells isolated from the mesenteric lymph nodes of mice infected with *Trichinella spiralis* [12]. Subsequently, it has been reported that IL-9 plays important roles in promoting epithelial paracellular permeability, mast cell proliferation, and isotype switching and in enhancing host protective immunity [13–15]. A recent study elucidated that the induction of antigen-specific Th9 immunity is significant for host defence against *Trichinella spiralis*. Experiments on combined transplantation of Th2 and Th9 cells showed stronger anthelminthic responsiveness than transplantation of Th2 cells alone [16]. Another report described that the exogenous IL-9 in IL-9 transgenic mice promoted worm shedding through goblet cell hyperplasia, increased muscle contraction, and upregulation of IL-4, MMCP1, and IL-13 [17]. It is worth mentioning that the different effects of IL-9 appear to be parasite dependent based on a comparison with the effects of IL-9 neutralization after infection with *Trichinella muris* [18]. In conclusion, compelling evidence clearly points to the protective and central roles of IL-9 and Th9 immune responses in the context of parasitic nematode infection [19–22].

In a previous study, we discovered the immunomodulatory effects of *H. contortus* excretory and secretory proteins (HcESPs) on Th9 cells, IL-9 production and the TGF-β/Smad signalling pathway. The production of Th9 cells in goats was significantly increased at 7, 10, 14, 18, 21 and 28 days post-infection (dpi) with *H. contortus*. During infection, the production of IL-9 and transcriptional levels of genes related to the TGF-β/Smad pathway gradually increased. The Th9 immune response was also reduced after deworming [23]. However, little is known about the exact molecule(s) in HcESPs that induce Th9 immune responses. The mechanism by which they induce Th9 immunity remains poorly understood. In this study, we investigated the molecules in HcESPs that bind with Th9 cells by flow cytometry, co-immunoprecipitation (Co-IP) and liquid chromatography tandem-mass spectrometry (LC–MS/MS). In addition, the immunomodulatory effects of two Th9 cell-binding proteins were verified. This study provides a better way to explore the immunostimulatory antigens of *H. contortus* and identifies candidates for the development of vaccines against *H. contortus*.

## Materials and methods

### Ethical declaration

The treatment of animals in our research was in conformity with the guidelines of the Animal Welfare Board of Nanjing Agricultural University, China. The protocols were approved by the Animal Ethics Committee of Nanjing Agricultural University, China. The experiments were authorized by the Science and Technology Agency of Jiangsu Province (Approval ID: SYXK (SU) 2010-0005).

### Parasites and animals

*Haemonchus contortus* was isolated in Nanjing and propagated in helminth-free goats as described previously [24]. In addition, the culture and collection of third-stage larva (L3) were carried out as previously described [25, 26].

Local healthy 5- to 7-month-old goats (*n* = 12) were fed in ventilated cages to prevent accidental nematode infections and provided alfalfa pellets, hay, and water at all times. All goats were dewormed by oral administration of albendazole following the manufacturer’s instructions (10 mg/kg). Faecal egg counts (FEC) were performed three times per week. Seven days later, no eggs were detected. Animals were randomly divided into three groups including the blank group (*n* = 5), challenge group (*n* = 5) and ESP-collecting group (*n* = 2). The blank group did not receive any treatment, while a dose of 8000 *H. contortus* L3 was orally administered to each goat in the challenge group and ESP-collecting group. The FEC assay was performed to confirm infection of the goats.

Wistar rats (body weight 180–220 g) were purchased from the Experimental Animal Center of Jiangsu, China, and were reared in the Animal Experimental Laboratory of Nanjing Agricultural University under aseptic conditions.

### Collection of HcESPs and preparation of polyclonal antibodies

Goats in the ESP-collecting group were euthanized at 35 dpi. Adult *H. contortus* were collected immediately from the abomasum following previously published procedures [27]. The pooled parasites (males and females) were washed with sterile phosphate-buffered saline (PBS) five times. Then, the worms were cultivated in Roswell Park Memorial Institute (RPMI) 1640 medium (100 worms/mL) (Gibco, Grand Island, New York, USA) containing penicillin (100 IU) and streptomycin (0.1 mg/mL; Pen strep, Gibco, USA) at 37 °C in an incubator containing 5% CO_2_. After 24 h of cultivation, the supernatant was collected and filtered through 0.22-μm filters. The ESPs were desalted and concentrated with a 3 kDa-cut-off centrifugal tube (Millipore, Bedford, MA, USA). The protein concentration (738.25 μg/mL) was determined by the BCA method [28]. The HcESPs were confirmed by 12% SDS–PAGE.

To produce polyclonal antibodies, 0.3 mg of HcESPs in Freund’s complete adjuvant (FCA; diluted 1:1, Sigma, USA) was administered to Wistar rats. After the primary immunization, the rats were boosted 4 times with the same dose of HcESPs with Freund’s incomplete adjuvant (FIA; diluted 1:1, Sigma, United States) at one-week intervals. An indirect enzyme-linked immunosorbent assay (ELISA) was carried out to determine the specific IgG titre in rat serum (1:2^20^) as previously described [29]. Sera collected from rats before vaccination were used as a negative control. All sera were stored at − 70 °C.

### Isolation of peripheral blood mononuclear cells (PBMCs) from goats

PBMCs were separated via a standard Ficoll-Paque protocol (GE Healthcare, Munich, USA) [30]. After 2 washes with sterile PBS (pH 7.4), the density of PBMCs was adjusted to 1 × 10^6^ cells/mL, and the cells were cultured in RPMI 1640 medium containing 10% foetal bovine serum (FBS; Thermo Fisher, USA), penicillin (100 IU) and streptomycin (0.1 mg/mL; Pen strep, Gibco, USA).

### Isolation of Th9 cells from goat PBMCs

A sorting method using flow cytometry was used to obtain Th9 cells in in vivo and in vitro experiments. The density of PBMCs from the blank group was adjusted to 1 × 10^6^ cells/mL, and the cells were cultured in RPMI 1640 medium supplemented with HcESPs (80 μg/mL, shown to be the best concentration in a previous study) for 48 h at 37 °C in a humidified 5% CO_2_ incubator. Then, the cells were stimulated with 10 μg/mL brefeldin A solution (BFA; BD Biosciences, San Jose, CA, USA) containing 10 ng/mL phorbol myristate acetate (PMA; Sigma–Aldrich, MO, USA) and 1 μg/mL ionomycin (Sigma–Aldrich, MO, USA) for another 4–6 h before intracellular staining [23]. Then, the cells were centrifuged at 500 × *g* for 10 min, and the pelleted cells were washed with PBS (pH 7.4) 3 times. Approximately 1 × 10^8^ cells were obtained and incubated with fluorescein isothiocyanate (FITC)-labelled anti-CD2 and Alexa Fluor 488-labelled CD4 antibodies (BD Biosciences, Becton, USA) in the dark at 4 °C for 30 min (following the manufacturer’s instructions). Subsequently, the cells were centrifuged at 500 × *g* for 5 min, collected, and treated with 500 μL of fixation buffer (BD Biosciences) at 4 °C for 20 min in the dark. Finally, the cells were permeabilized by adding BD Perm/Wash buffer (BD Biosciences, Becton, USA) and stained with PE-Cy5-labelled anti-IL-9 (GenScript, New Jersey, USA) and PE-labelled anti-IL-10 antibodies (BD, Pharmingen, USA) for 30 min. The stained cells were suspended in 500 µL of PBS and analysed with a BD FACSAria II SORP (BD Biosciences) [31]. The Th9 cell population was defined by the CD2 + CD4 + IL-9 + IL-10 + phenotype. For the in vivo study, Th9 cells were isolated from goats in the challenge group at 7, 15, 35 and 50 dpi as stated above, and the goats in the blank group were used as negative controls.

### Co-IP test

Sorted Th9 cells from the blank group incubated with ESPs and those isolated from the challenge group at 7, 15, 35 and 50 dpi were washed with PBS three times and lysed with RIPA lysis buffer (Thermo Fisher Inc., Rockford, IL, USA) containing 1% phosphatase and protease inhibitors (Thermo Fisher Inc., Rockford, IL, USA). The cell lysates were centrifuged at 12 000 *g* for 10 min at 4 °C, followed by incubation with 2.0 μg of normal rat IgG and 40 μL of Protein A/G PLUS-Agarose (Santa Cruz Biotechnology, Texas, USA) at 4 °C for 30 min to remove nonspecific binding proteins. To precipitate the bound HcESPs, the cell lysates were incubated with anti-HcESPs rat IgG at 4 °C overnight. Then, the samples were deposited using 20 μL of Protein A/G PLUS-Agarose and washed 4 times. To confirm that the HcESPs bound to Th9 cells were collected properly, the samples obtained by immunoprecipitation were evaluated by 12% SDS–PAGE and Western blot analysis using anti-HcESPs rat IgG as the primary antibody and normal rat IgG as the negative control.

### Shotgun LC–MS/MS analysis

Samples collected by Co-IP were digested with trypsin and analysed by shotgun LC–MS/MS using a Q Exactive instrument (Thermo Finnigan, San Jose, CA, USA) at Shanghai Bio Profile Technology Company Ltd. (Shanghai, China) as previously described [32]. For proteomic identification, based on the corresponding UniProt database (UniProt_*Haemonchus_contortus*_4037_20191119.fasta), Mascot software 2.2 was used for peptide mass fingerprinting and peptide sequence tagging (v.2.2, Matrix Science, London, UK). Carbonamidomethyl (C) was identified as a fixed modification, and oxidation (M) was identified as a variable modification, allowing less than two missed cleavages. Furthermore, all the identified peptides were screened with a false discovery rate (FDR) ≤ 0.01 and filtered for a score ≥ 20. The peptide mass tolerance was 20 ppm, and the fragment mass tolerance was 0.1 Da. Protein identification in the MASCOT search was applied to UniProtKB and QuickGO. Gene Ontology (GO) classification (molecular function, biological process and cellular component terms) was carried out using Blast2GO based on the BLASTP results.

### Cloning genes encoding *H. contortus* DNA/RNA helicase domain containing protein (HcDR) and GATA transcription factor (HcGATA)

To confirm the effects of the identified proteins on Th9 cells and IL-9 expression, the 2 proteins DNA/RNA helicase domain-containing protein (HcDR) and GATA transcription factor (HcGATA) were selected for further investigation.

Total RNA was extracted from adult *H. contortus* worms using TRIzol reagent (Invitrogen, New York, USA) [33]. The isolated RNA (OD260/280 = 1.99, 1421.26 ng/μL) was reverse transcribed with a cDNA synthesis kit (TaKaRa Biotechnology, Dalian, China). The genes encoding HcDR and HcGATA were amplified by reverse transcription PCR (RT–PCR) using specific primers (Additional file 1) based on the sequences of HcDR (EBI No. CDL95557.1) and HcGATA (AAQ18783.1). The PCR products were ligated into the pMD-19 T vector (TaKaRa, Japan) to produce pMD19T-HcDR and pMD19T-HcGATA, respectively, followed by endonuclease cleavage and sequence analysis (SnapGene 4.3.7, USA; Additional file 1). The ORF was sub-cloned into a prokaryotic expression vector (pET-28a (+) or pET-32a (+) (Novagen, USA) to generate recombinant HcDR or HcGATA.

### Expression and purification of recombinant HcDR and HcGATA

The expression of the recombinant HcDR and HcGATA proteins was performed as described previously [34]. In brief, *E. coli* BL21 (DE3) cells transfected with the plasmid pET-28a-HcDR or pET-32a-HcGATA were incubated at 37 °C until the OD600 was in the range of 0.6–0.8. Isopropyl-β-d-thiogalactopyranoside (IPTG) was added to induce protein expression. The His-tagged recombinant proteins were purified with a His Trap FF kit (GE Healthcare, USA) following the manufacturer’s protocol.

### Effects of recombinant HcDR and HcGATA on Th9 cells and IL-9 expression in vitro

To estimate the effects of recombinant HcDR and HcGATA on Th9 cells, PBMCs were harvested from the blank group and incubated with various concentrations of rHcDR or rHcGATA (5, 10, 20, 40 and 60 μg/mL) in vitro in 12-well culture plates containing RPMI 1640 medium for 12 h at 37 °C with 5% CO_2_. Subsequently, the method was same as “isolation of Th9 cells from goat PBMCs”.

To detect the transcriptional level of IL-9 in vitro, PBMCs from the blank group were incubated with various concentrations of rHcDR or rHcGATA (5, 10, 20, 40, 60 μg/mL) in vitro in 24-well culture plates containing RPMI 1640 medium for 24 h at 37 °C. Subsequently, RNA was extracted from these cells using a Total RNA Kit (Omega, USA) according to the manufacturer’s instructions. The mRNA expression of IL-9 was assessed as described elsewhere [28]. Specific primers for the β-actin gene (endogenous reference) and target gene IL-9 (F: 5′-GATGCGGCTGATTGTTT-3′, R: 5′-CTCGTGCTCACTGTGGAGT-3′) were used, and the relative transcriptional levels of IL-9 were normalized to those of β-actin based on the 2^−∆∆Ct^ method [23]. Real-time PCR was performed, and the data were recorded with an ABI 7500 (Applied Biosystems, USA).

### Statistical analysis

GraphPad Prism 7.0 (GraphPad Prism, USA) was used for statistical analyses. All data obtained in this study are shown as the mean ± SD. One-way ANOVA followed by Tukey’s post-hoc test was employed to compare multiple groups, and results were considered statistically significant at *p < 0.05, **p < 0.01, and ***p < 0.001. Flow cytometry data were analysed using Flow Jo software (version 10, USA).

## Results

### HcESP collection and anti-HcESP rat IgG generation

**Figure 1:**
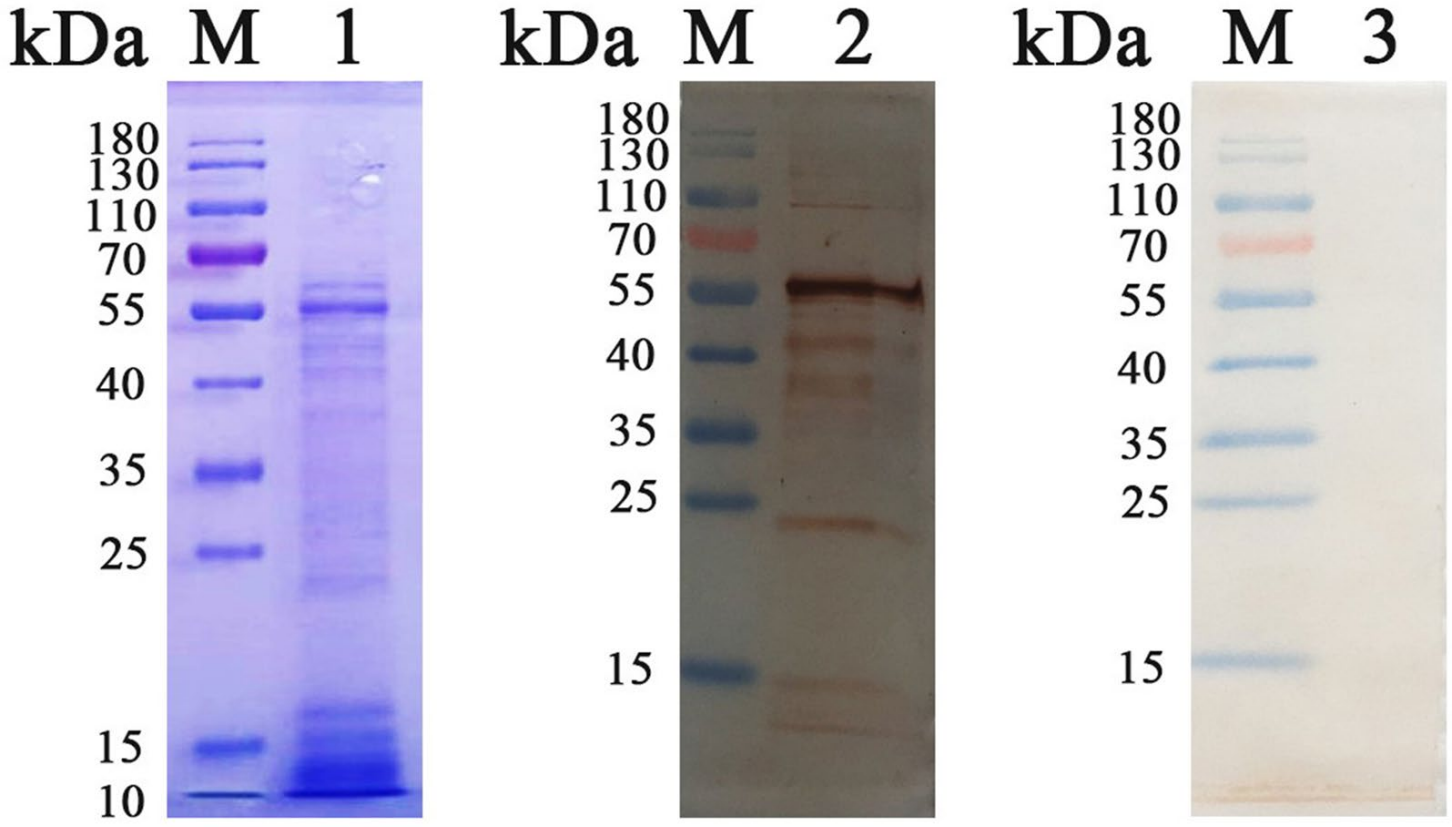
**Collection of HcESPs and preparation of anti-HcESPs rat IgG.** Lane 1: SDS–PAGE showing HcESP production; Lanes 2, 3: Western blot showing HcESPs identified with anti-HcESPs rat IgG as the primary antibody and normal rat IgG as the negative control.

HcESPs were isolated with 12% SDS–PAGE gels. Coomassie brilliant blue staining indicated that the molecular weight of the HcESPs ranged from 10 to 180 kDa (Figure 1, Lane 1). Western blot analysis showed that the naturally distributed HcESPs ranging from 10 to 180 kDa could be recognized by anti-HcESP rat IgG (Figure 1, Lane 2), while control normal rat IgG did not recognize any band (Figure 1, Lane 3). This showed that anti-HcESP rat IgG was specific to HcESPs.

### Sorting of Th9 cells in in vivo and in vitro experiments

**Figure 2:**
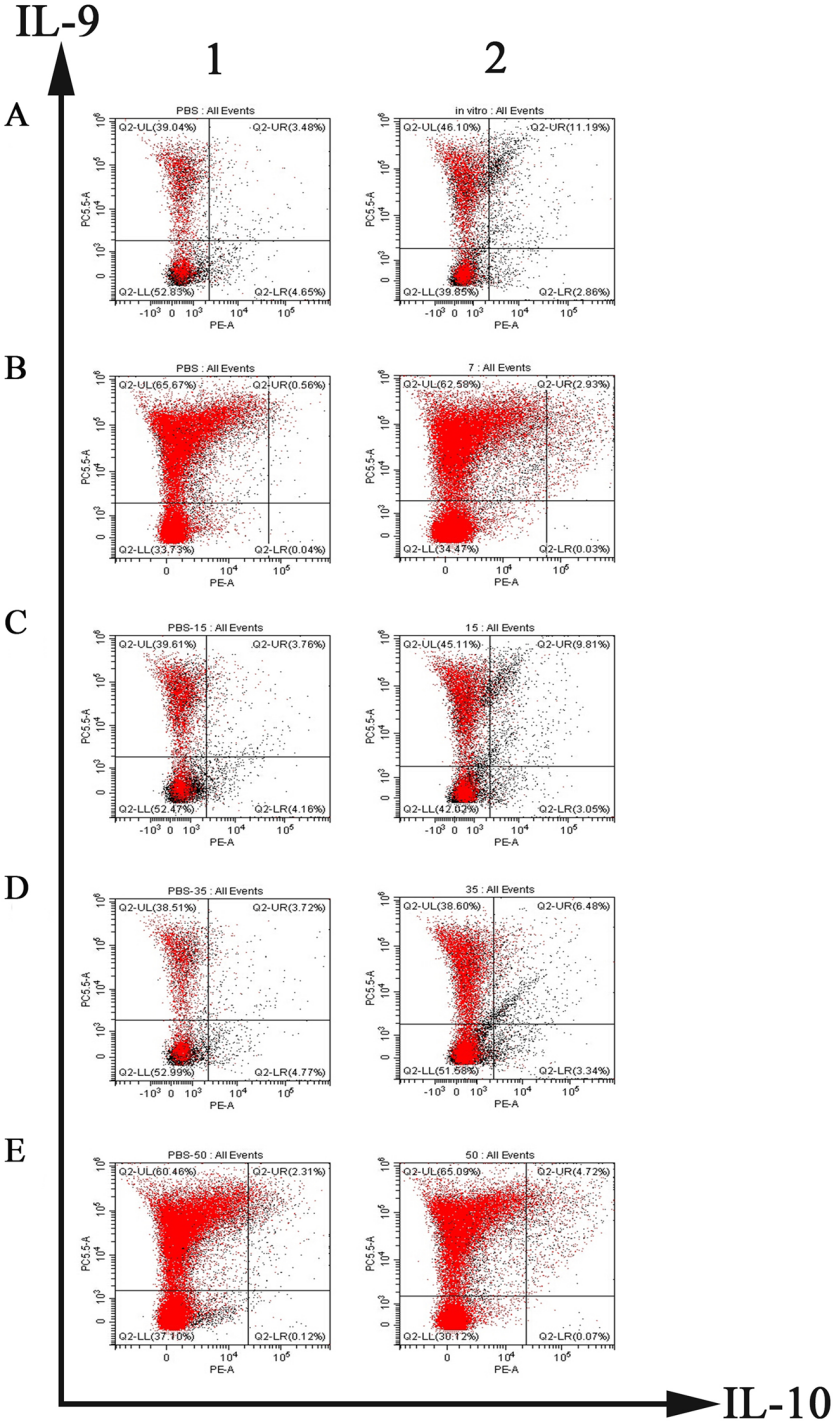
**Th9-cell sorting by flow cytometry**. **A** Th9 cells induced by HcESPs in vitro. PBMCs were treated with PBS (0 μg/mL HcESPs as the control) (**A** Panel 1) or 80 μg/mL HcESPs (**A** Panel 2). Th9 cells were sorted by flow cytometry using CD2 + CD4 + IL-9 + IL-10 + as the gate. **B**–**E** Percentages of Th9 cells at 7 (**B**), 15 (**C**), 35 (**D**) and 50 (**E**) dpi. Samples were collected from the blank group (**B–E** Panel 1) and challenge group (**B–E** Panel 2) at 7, 15, 35 and 50 dpi.

To evaluate HcESP binding to Th9 cells, flow cytometry was performed with gating on CD2^+^CD4^+^ T cells using anti-IL-9 and anti-IL-10 antibodies for intracellular staining to count and sort Th9 cells. An in vitro study revealed that the production of Th9 cells in vitro was 11.19% (Figure 2A, Panel 2) and that of untreated cells was 3.48% (Figure 2A, Panel 1). The results showed that 80 μg/mL HcESPs facilitated Th9-cell differentiation compared to no treatment.

To monitor the dynamics of Th9-cell generation during *H. contortus* infection, PBMCs were isolated directly from challenged goats. The results showed that the percentages of Th9 cells at 7, 15, 35 and 50 dpi were 2.93% (Figure 2B, Panel 2), 9.81% (Figure 2C, Panel 2), 6.48% (Figure 2D, Panel 2), and 4.72% (Figure 2E, Panel 2), respectively, while those in the blank group were 0.56% (Figure 2B, Panel 1), 3.76% (Figure 2C, Panel 1), 3.72% (Figure 2D, Panel 1), and 2.31% (Figure 2E, Panel 1), respectively.

### Co-IP assay

**Figure 3:**
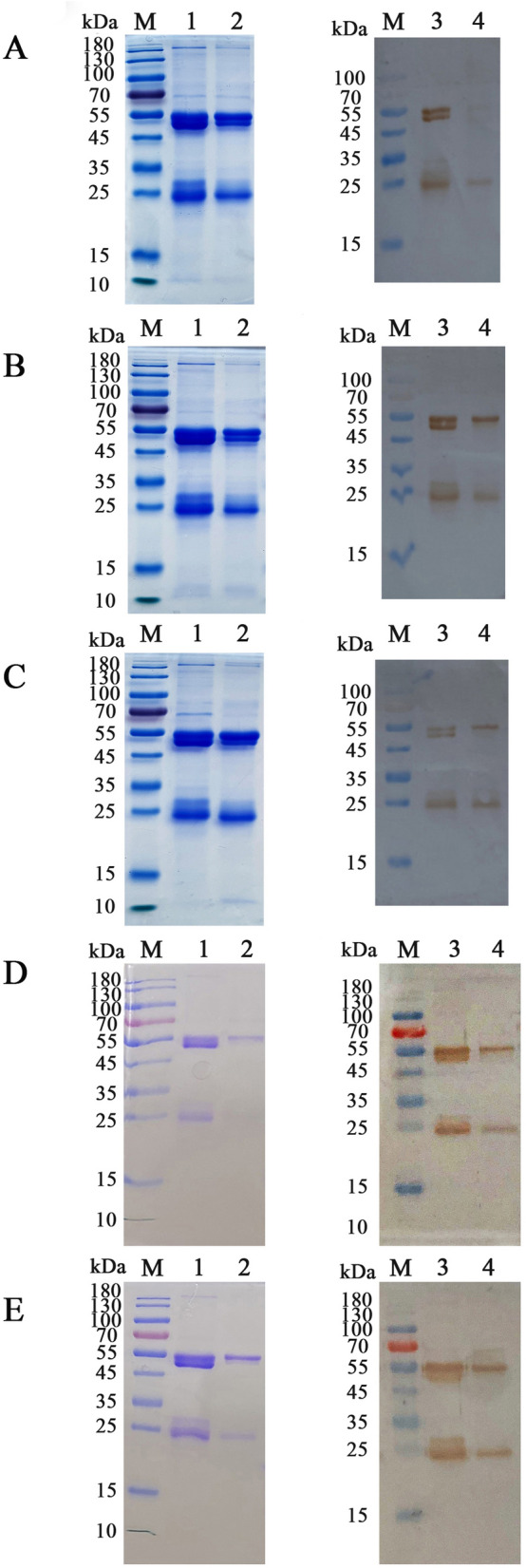
**Co-IP assays indicated that HcESPs bind to Th9 cells**. Lane M: marker. Lanes 1 and 2: SDS–PAGE showing that cell lysates were immunoprecipitated by anti-HcESPs rat IgG (Lane 1) or normal rat IgG as a negative control (Lane 2). Lanes 3 and 4: Western blot showing that cell lysates were immunoprecipitated by anti-HcESPs rat IgG (Lane 4) or normal rat IgG as a negative control (Lane 4). Cell lysates from in vitro experiments (**A**) or in vivo experiments collected at 7 (**B**), 15 (**C**), 35 (**D**) and 50 (**E**) dpi. Anti-ESPs rat IgG was used in the Western blot analysis to identify proteins that bound to Th9 cells.

Complexes (Th9 cell protein bound to HcESPs) were extracted using anti-HcESPs rat IgG (Figures 3A–E, Lane 1) or normal rat IgG (Figures 3A–E, Lane 2) as the IP antibody. Western blot analysis showed that a variety of proteins were detected in the complexes, with the bands ranging from 20 to 180 kDa in vitro (Figure 3A, Lane 3). Similar results were found at 7 (Figure 3B, Lane 3), 15 (Figure 3C, Lane 3), 35 (Figure 3D, Lane 3) and 50 (Figure 3E, Lane 3) dpi for the in vivo study. Although the heavy and light chains of normal rat IgG could be detected, normal rat IgG did not pull down any proteins producing bands corresponding to the complexes (Figures 3A–E, Lane 4). These observations suggest that some HcESPs interact with host Th9 cells in vitro and in vivo during *H. contortus* infection.

### Proteins identified by LC–MS/MS analysis

The EMBL-EBI database with the Mascot search engine was used to search our obtained LC–MS/MS datasets. Each sample was independently searched. In the in vitro experiment, 40 proteins were identified (Additional file 2, in vitro). For the in vivo study, 38 proteins were identified at 7 dpi (Additional file 2, 7 dpi), 47 at 15 dpi (Additional file 2, 15 dpi), 42 at 35 dpi (Additional file 2, 35 dpi), and 142 at 50 dpi (Additional file 2, 50 dpi).

**Figure 4:**
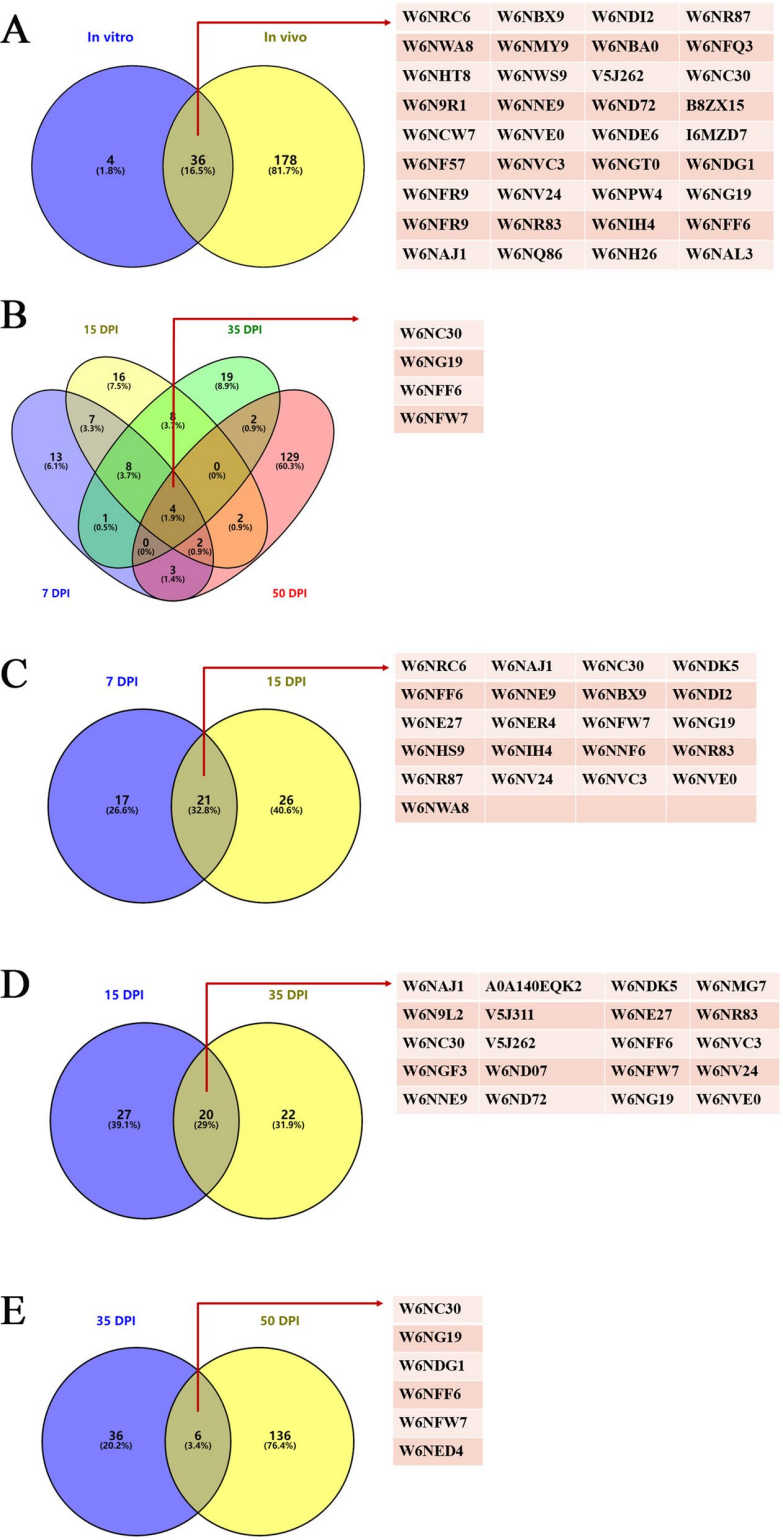
**Venn diagram of binding proteins. A** The binding proteins shared between in vitro and in vivo experiments. **B** The binding proteins shared among different developmental stages (7, 15, 35 and 50 dpi). **C** The binding proteins shared between 7 and 15 dpi. **D** The binding proteins shared between 15 and 35 dpi. **E** The binding proteins shared in the in vivo experiment between 35 and 50 dpi.

There were 36 shared proteins between the in vivo and in vitro experiments (Figure 4A, Additional file 3). Four proteins were shared among 7, 15, 35 and 50 dpi in the in vivo experiments (Figure 4B, Additional file 4). A total of 21 proteins were identified at 7 and 15 dpi, including peptidase S28 domain containing protein, immunoglobulin I-set and fibronectin and serine threonine protein kinase-related domain containing protein, and inner centromere protein domain containing protein (Figure 4C, Additional file 5). Twenty shared proteins were found for 15 and 35 dpi, such as RNA-directed DNA polymerase, reverse transcriptase domain-containing protein, aminopeptidase, nematode cuticle collagen, collagen triple helix repeat domain-containing protein, and zinc finger domain-containing protein (Figure 4D, Additional file 6). In addition, 6 proteins were shared between 35 and 50 dpi, including bardet-Biedl syndrome 1 protein isoform 2 and some other uncharacterized proteins (Figure 4E, Additional file 7).

### Ontological analysis of recognized proteins

The GO signatures of 218 proteins identified in vivo and in vitro were available in the database (Additional file 8). The proteins were categorized by their molecular function, biological process, and cellular component terms in compliance with the GO hierarchy utilizing the Web Gene Ontology Annotation Plot.

**Figure 5:**
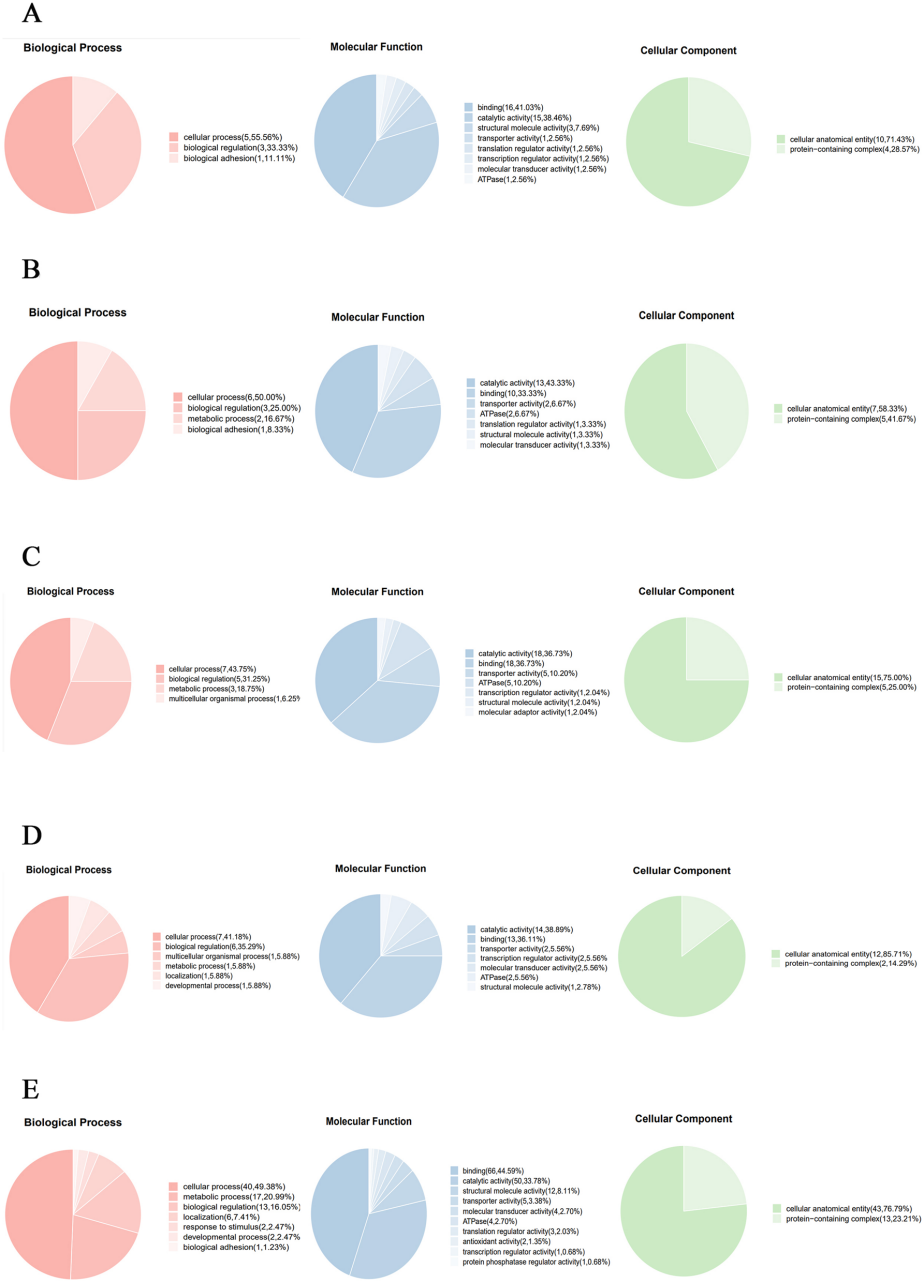
**GO annotation**. **A** HcESPs that bound to goat Th9 cells in vitro. **B** HcESPs identified at 7 dpi. **C** HcESPs identified at 15 dpi. **D** HcESPs identified at 35 dpi. **E** HcESPs identified at 50 dpi.

Among the 40 proteins identified in vitro, 39 were allocated to 8 molecular function subcategories, with the main ones being catalytic and binding actions. Additionally, 3 proteins were assigned to biological process terms, including biological adhesion, biological regulation, and cellular process. For the cellular component category, 2 cellular component terms were allocated to 14 identified proteins, including protein-containing complex and cellular anatomical entities (Figure 5A, Additional file 9, in vitro).

The GO annotation of the interacting proteins identified at 7, 15, 35 and 50 dpi is presented in Figures 5B–E. Most of the proteins were assigned to 7 molecular function terms, with binding and catalytic actions as the two main subcategories. Most of the interacting proteins were allocated to 4 biological process terms, including biological adhesion, biological regulation, cellular process, and metabolic process. In addition, the identified proteins were assigned to 2 cellular component terms, protein-containing complex and cellular anatomical entities.

Consistent with previous proteomic analyses [32, 35, 36], serine-threonine/tyrosine protein kinases were enriched in the protein kinase activity subcategory in this study. Protein kinases are key regulators of cellular functions and are particularly prominent in the signal transduction and coordination involved in protein–protein interactions, inflammation, cell survival and the cell cycle [37, 38]. In particular, most identified ESPs were enriched in binding activity terms, namely, ion binding, nucleic acid binding, ATP/GTP binding, unfolded protein binding, peptide binding and DNA/RNA binding. Proteins involved in these functions are normally associated with energy metabolism, signalling, and transcription [36–38]. As external stimuli, these exogenous proteins may bind to host Th9 cells, interfere with the intracellular homoeostatic balance and disrupt many cellular functions.

### Effects of rHcDR on Th9 cells and IL-9 expression in vitro

**Figure 6:**
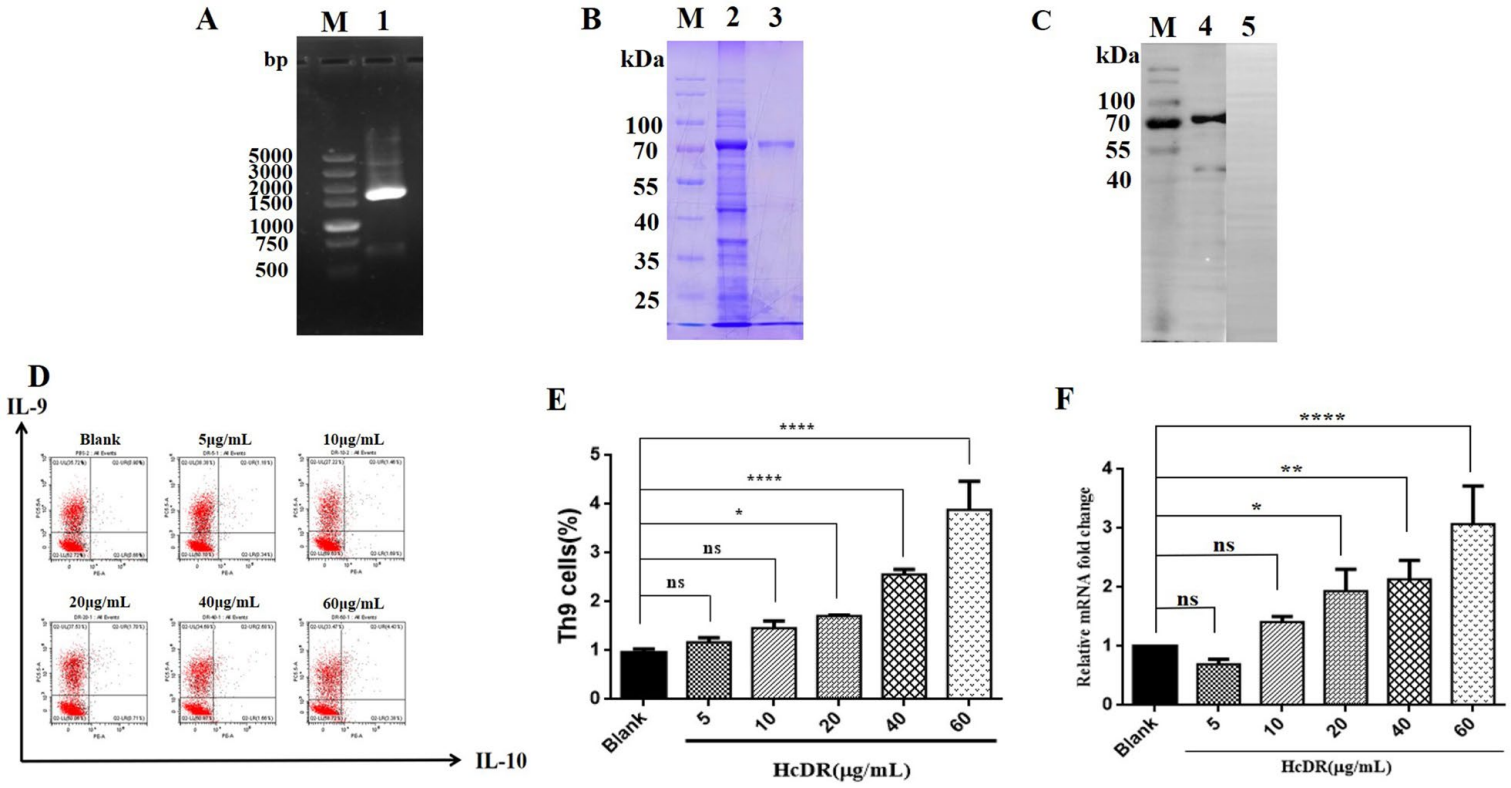
**Effects of rHcDR on Th9 cells and IL-9 expression in vitro. A** Agarose gel electrophoresis of the HcDR gene. Lane 1: amplification products of the HcDR gene. Lane M: DNA molecular weight marker. **B** Purification of rHcDR. Lane 2: before purification. Lane 3: purified rHcDR. Lane M: standard protein molecular marker. **C** Western blot. Lane 4: rHcDR recognized by serum from an *H. contortus*-infected goat. Lane 5: No recognition by normal serum. Lane M: standard protein molecular marker. **D** The effects of rHcDR on Th9-cell proliferation. Goat PBMCs were treated with different concentrations of rHcDR (0, 5, 10, 20, 40 and 60 μg/mL). Th9 cells were detected by flow cytometry using staining with antibodies specific for typical intracellular cytokines (IL-9 and IL-10). **E** Proportions of Th9 cells observed with different concentrations of rHcDR (0, 5, 10, 20, 40 and 60 μg/mL). Data are presented as the mean ± SD and are representative of triplicate experiments (*p < 0.05, ****p < 0.0001). **F** Fold change in relative IL-9 mRNA expression. Goat PBMCs were stimulated with different concentrations of rHcDR. The significance level was set at *p < 0.05, **p < 0.01, or ****p < 0.0001, and “ns” indicates non-significance compared with the control (blank). Data are representative of three independent experiments.

The ORF of HcDR (1695 bp in size) (Figure 6A) was inserted into the pMD-19 T vector. Sequence analysis showed 100% similarity with the sequence reported in EBI. SDS–PAGE indicated that recombinant HcDR was expressed, with observation of the predicted band of approximately 66.15 kDa (Figure 6B). Western blotting also showed that rHcDR (Figure 6C) was recognized by sera from *H. contortus*-infected goats.

To estimate the effects of rHcDR on Th9 cells, different concentrations of rHcDR were incubated with goat PBMCs, and the population of Th9 cells was evaluated. Flow cytometry results revealed that 60 μg/mL rHcDR induced the highest production of Th9 cells, achieving a percentage of 4.43% (Figure 6D). Treatment with 5, 10, 20, or 40 μg/mL rHcDR also promoted the generation of Th9 cells, achieving proportions of 1.18%, 1.46%, 1.70% and 2.68%, respectively (Figure 6D). The results showed that treatment with rHcDR (20–60 μg/mL) significantly stimulated Th9-cell proliferation compared with control treatment (0 μg/mL, 0.9%) (Figure 6E).

qPCR assays were performed to assess IL-9 transcription after treatment with different concentrations of rHcDR. The relative mRNA expression in the groups treated with 20, 40 or 60 μg/mL rHcDR was increased by 1.93- (*p* < 0.05), 2.14- (*p* < 0.01) and 3.07-fold (*p* < 0.001), respectively, when compared with that in the blank control group (PBS, fold = 1), while that in the groups treated with 5 μg/mL (fold = 0.69, *p* > 0.05) or 10 μg/mL (fold = 1.41, *p* > 0.05) did not show significant differences when compared with that in the blank group (Figure 6F).

### Effects of rHcGATA on Th9 cells and IL-9 expression in vitro

**Figure 7:**
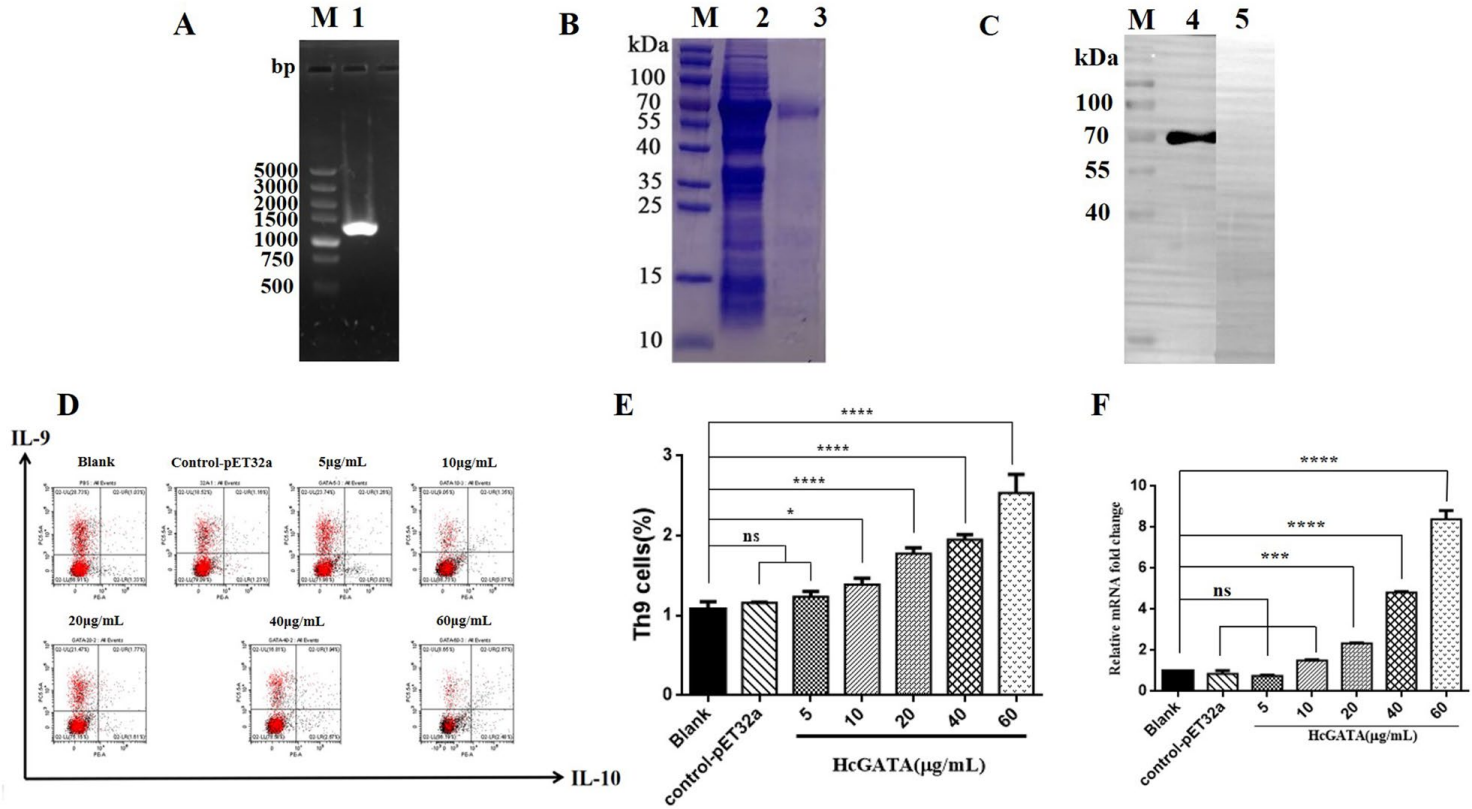
**Effects of rHcGATA on Th9-cell proliferation and IL-9 transcription. A** Agarose gel electrophoresis of the HcGATA gene. Lane 1: reverse transcription PCR products of HcGATA; Lane M: DNA molecular weight marker. **B** Purification of rHcGATA. Lane 2: rHcGATA before purification. Lane 3: purified rHcGATA. Lane M: standard protein molecular marker. **C** Western blot. Lane 4: rHcGATA protein recognized by serum from an *H. contortus*-infected goat. Lane 5: rHcGATA was not recognized by normal serum. Lane M: standard protein molecular marker. **D** The effects of HcGATA on the proliferation of Th9 cells in vitro. PBMC-derived Th9 cells treated with a control (0 μg/mL) or different concentrations of HcGATA (5, 10, 20, 40, 60 μg/mL) were tested by flow cytometry using antibodies specific for typical intracellular cytokines (IL-9 and IL-10). **E** Proportions of Th9 cells observed with different concentrations of HcGATA (0, 5, 10, 20, 40 and 60 μg/mL). Data are presented as the mean ± SD representative of triplicate experiments (ns* p* > 0.05, *p < 0.05, ****p < 0.0001). **F** Fold change in relative IL-9 mRNA expression. Goat PBMCs were stimulated with different concentrations of rHcGATA. The significance level was set at ***p < 0.001, or ****p < 0.0001, and “ns” indicates non-significance compared with the control group. Data are representative of three independent experiments.

The ORF of HcGATA was 1254 bp (Figure 7A). Sequence analysis showed 100% similarity with the sequence reported in EBI. SDS–PAGE results revealed that rHcGATA was expressed in *E. coli*, producing a band of approximately 63.98 kDa (Figure 7B). Western blot analysis showed that rHcGATA (Figure 7C) could be recognized by sera from *H. contortus*-infected goats.

Different concentrations of rHcGATA were incubated with goat PBMCs, and the results showed that 60 μg/mL rHcGATA had the greatest capacity to stimulate Th9-cell production (2.67%). The percentages of Th9 cells were recorded to be 1.26%, 1.35%, 1.77% and 1.94% when goat PBMCs were treated with 5, 10, 20 or 40 μg/mL rHcGATA, respectively (Figure 7D). Flow cytometry data showed that treatment with more than 10 μg/mL rHcGATA markedly stimulated Th9-cell proliferation compared with control treatment (0 μg/mL, 1.03%) (Figure 7E).

The transcriptional level of IL-9 in the groups treated with 20, 40 or 60 μg/mL rHcGATA was increased 2.34- (*p* < 0.001), 4.82- (*p* < 0.001) and 8.39-fold (*p* < 0.001) when compared with that in the blank group (PBS, fold = 1), while that in the groups treated with 5 μg/mL (fold = 0.75, *p* > 0.05) or 10 μg/mL (fold = 1.50, *p* > 0.05) did not show a significant difference when compared with that in the blank group. Furthermore, no differences were found between the blank and pET-32a controls (fold = 0.86, *p* > 0.05) (Figure 7F).

## Discussion

The regulation of T helper cell immune responses is accomplished through the secretion of specific cytokines. Together with master regulatory transcription factors, these cytokines define subsets of Th cells and their unique functions and properties [39–42]. IL-9 is mainly produced by Th9 cells but also by type 2 innate lymphoid cells (ILC2s), Th17 cells, CD8+ cells, mast cells, Th2 cells, NK cells, and Treg cells under certain conditions [43–46]. Although many cells can produce IL-9, Th9 cells also produce IL-10 while serving as the main source of IL-9 and do not produce other cytokines, such as IL-4, IL-5, and IL-13. This pattern separates Th9 cells from other IL-9-producing cells [47]. Therefore, in our study, the CD2 + CD4 + IL-9 + IL-10 + phenotype was selected as the criterion for screening Th9 cells. In addition, since there is currently no literature on the physiological dose range of HcESPs and more Th9 cells were preferred in our study, we chose to incubate PBMCs with 80 μg/mL ESPs based on previous studies proving that more than 20 μg/mL ESPs could significantly increase the number of Th9 cells in vitro.

Nematodes can secrete ESPs to control and modulate host immune responses. More importantly, ESPs have strong immunogenicity and can be used as candidate molecules for vaccine research [48]. In recent decades, a significant amount of research has been performed on the composition and function of HcESPs to improve the prevention and treatment of *H. contortus* infection. Some HcESPs have been shown to modulate the host immune response [27]. In our study, we identified a total of 218 HcESPs interacting with goat Th9 cells in vitro and in vivo at different developmental stages by Co-IP and LS–MS/MS. This is the first time HcESPs binding to Th9 cells in vitro and in vivo (from L4 to adulthood in a goat host) was reported. Notably, nematode cuticle collagen and collagen triple helix repeat domain-containing proteins were identified among the interacting ESPs both in vitro and in vivo. Collagen is a major component of the multi-layered structure in the nematode cuticle. Nematode cuticle collagen and collagen triple helix repeat domain-containing protein are secreted to support parasite immune escape by regulating the host immune response.

Pleckstrin homology (PH) domain protein and Dbl homology (DH) domain-containing protein were also identified in vitro and in vivo. The PH domain, which is involved in cell signalling and cytoskeletal rearrangement, is a small protein module of approximately 120 amino acids found in many proteins. Recent studies have shown that a subregion of the PH domain can identify the products of agonist-stimulated phosphoinositide 3-kinase (PI3K) [49, 50]. PI3K is involved in the signal transduction and activation of immune cells. In addition, PI3K activates p21-activated kinase 1 (PAK1) at the plasma membrane by promoting casein kinase 2 (CK2)-mediated phosphorylation of PAK1 through CKIP-1, one of the pleckstrin homology domain-containing proteins [51]. PAK1 is a major downstream effector of the GTPases Cdc42 and Rac1 in the Rho family and plays important roles in the regulation of cell morphology and motility. TgPH1 of *Toxoplasma gondii* is a protein predominantly formed by the preferential binding of the pleckstrin homology (PH) domain to phosphoinositide phosphatidylinositol-3,5-bisphosphate (PI(3,5) p2) [52]. The Dbl protein family includes guanine nucleotide exchange factors (GEFs), which can catalyse the exchange of guanine nucleotides to promote the formation of active GTP-binding proteins; the active proteins in turn activate Rho family GTPases [53, 54]. Rho family GTPases function as GDP/GTP switches regulated similarly to Ras. Upon activation, the Rho GTPases RhoA, Rac1 and Cdc42 can regulate actin cytoskeleton organization, gene expression and cell cycle progression by interacting with multiple effectors [55, 56]. It has been reported that the DH domain not only has the same function as the Rho protein activator but also contributes to the coupling of cellular signalling and Ras activation [57, 58]. Pleckstrin homology (PH) domain protein and Dbl homology (DH) domain-containing protein have regulatory effects on Rho protein signal transduction, suggesting that these two proteins may play an important role in triggering the Th9 immune response in goats.

Peptidase M14 domain-containing protein was identified in early adulthood. Current studies on the peptidase family support the conclusion that peptides play a critical role in globin catabolism through haemoglobin cleavage [59]. Glyceraldehyde-3-phosphate dehydrogenase (GAPDH) was identified during later stages of adulthood. GAPDH is a glycolytic enzyme with multiple functional attributes [60]. It can bind to the complement factor C3, a key protein in the complement cascade, to regulate innate immune responses. Studies have shown that GAPDH-specific antibodies were found in some animals infected with *H. contortus* [61]. Therefore, GAPDH may be a potential drug candidate, and clinical trials have demonstrated partial protection against *H. contortus* after immunization with a GAPDH-specific DNA vaccine [62].

In this study, 2 of the Th9 cell-binding HcESPs were validated to induce Th9 immune responses. DNA/RNA helicase domain-containing protein was identified in vitro. Helicases play important roles in DNA replication, DNA repair, recombination, transcription, and translation and are involved in ribosome biogenesis, export, decay, RNA turnover, surveillance, translation, storage, and pre-mRNA processing during RNA metabolic processes. Various helicases have been identified to be involved in specific regulatory processes, some of which are indispensable for parasite survival and growth [63, 64]. Previously, some reports indicated that full-length helicases were found in the Plasmodium genome and that several helicases were identified to be produced by the parasite [63, 65–69]. Furthermore, helicases encoded by bacteria, viruses, and human cells have been extensively studied as targets for anticancer drugs, antibiotics, and new antiviral drugs [70]. GATA transcription factor was identified at 15 dpi. Transcription factors are essential in regulatory mechanisms and are increasingly being recognized as potential novel drug targets [71, 72]. The *Caenorhabditis elegans* GATA transcription factor elt-2 is involved in the regulation of endodermal differentiation and lineage-specific gene expression [73, 74]. In this study, the data from flow cytometry and qPCR assays showed that treatment with more than 20 μg/mL or 10 μg/mL rHcDR/HcGATA markedly stimulated Th9-cell generation and IL-9 expression compared to control treatment. These results indicated that these two proteins could induce Th9-cell generation and increase the level of IL-9 expression in vitro, but whether they can induce the Th9 immune response in vivo needs to be further studied.

In conclusion, 218 HcESPs binding to goat Th9 cells were identified, and two of them were confirmed to stimulate the goat Th9 immune response. Our data provide a baseline for better understanding the interactions between *H. contortus* and the host. However, the functions and regulatory mechanisms of HcESPs that bind to Th9 cells remain to be further investigated.

## Supplementary Information

**Additional file 1. Oligonucleotide primer sequences used for HcDR and HcGATA.**

**Additional file 2. Th9 cell-binding proteins identified by LC–MS/MS.** In vitro: the proteins identified from the in vitro study. 7 dpi: the proteins identified from a goat infected with *H. contortus* at 7 dpi. 15 dpi: the proteins identified from a goat infected with *H. contortus* at 15 dpi. 35 dpi: the proteins identified from a goat infected with *H. contortus* at 35 dpi. 50 dpi: the proteins identified from a goat infected with *H. contortus* at 50 dpi.

**Additional file 3. Shared proteins between in vivo and in vitro experiments.**

**Additional file 4. Shared proteins among different developmental stages (7, 15, 35 and 50 dpi).**

**Additional file 5. Shared proteins between 7 and 15 dpi.**

**Additional file 6. Shared proteins between 15 and 35 dpi.**

**Additional file 7. Shared proteins between 35 and 50 dpi.**

**Additional file 8. GO annotation of Th9 cell-binding proteins based on the cellular component, biological process and molecular function categories.**

**Additional file 9. GO annotation of Th9 cell-binding proteins based on the cellular component, biological process and molecular function categories.** In vitro: the proteins identified from the in vitro study. 7 dpi: the proteins identified from a goat infected with *H. contortus* at 7 dpi. 15 dpi: the proteins identified from a goat infected with *H. contortus* at 15 dpi. 35 dpi: the proteins identified from a goat infected with *H. contortus* at 35 dpi. 50 dpi: the proteins identified from a goat infected with *H. contortus* at 50 dpi.

**Additional file 10. The sequences of HcDR and HcGATA.**

## Data Availability

The datasets supporting the conclusions of this article are included in Additional files 1, 2, 3, 4, 5, 6, 7, 8, 9, 10.

## Abbreviations

ESPs: excretory and secretory proteins
HcDR: *H. contortus* DNA/RNA helicase domain containing protein
HcGATA: *H. contortus* GATA transcription factor
L3: third-stage larva
rHcDR: recombinant HcDR
rHcGATA: recombinant HcGATA
PBS: phosphate-buffered saline
IL: interleukin
PBMC: peripheral blood mononuclear cell
