# Supplementary material for: Identification of excretory and secretory proteins from Haemonchus contortus inducing a Th9 immune response in goats
Source: Vet Res. 2022 May 21;53:36. doi: 10.1186/s13567-022-01055-8 (PMC9123704; doi:10.1186/s13567-022-01055-8)
Supplement: Supplementary file 1 — Additional file 1. Oligonucleotide primer sequences used for HcDR and HcGATA. [file 13567_2022_1055_MOESM1_ESM.docx]

**Additional file 1 Oligonucleotide primer sequences used for HcDR and HcGATA application**

| Gene Name | Abbreviation | Protein ID | Specific primers | Enzyme |
| --- | --- | --- | --- | --- |
| GATA transcription factor | GATA | Q7YUE7 | CGC GGATCC ATGGAGAATTCTCACGATAGCC | *BamHI* |
|  |  |  | TT GCGGCCGC TCAAACATGTGGTGATCTTTCAGC | *Not I* |
| DNA RNA helicase domain containing protein | DR | W6NTY7 | CCC AAGCTTCC ATGCGTGTCGACGTTCAGAAG | *HindIII* |
|  |  |  | ATTT GCGGCCGC TTATTTTCTCGTCTTTTGCTTG | *Not I* |
